# Biglycan expression in the melanoma microenvironment promotes invasiveness via increased tissue stiffness inducing integrin-β1 expression

**DOI:** 10.18632/oncotarget.17160

**Published:** 2017-04-17

**Authors:** Hana Andrlová, Justin Mastroianni, Josef Madl, Johannes S. Kern, Wolfgang Melchinger, Heide Dierbach, Florian Wernet, Marie Follo, Kristin Technau-Hafsi, Cristina Has, Venugopal Rao Mittapalli, Marco Idzko, Ricarda Herr, Tilman Brummer, Hendrik Ungefroren, Hauke Busch, Melanie Boerries, Andreas Narr, Gabriele Ihorst, Claire Vennin, Annette Schmitt-Graeff, Susana Minguet, Paul Timpson, Justus Duyster, Frank Meiss, Winfried Römer, Robert Zeiser

**Affiliations:** ^1^ Department of Hematology and Oncology, University Medical Center, Faculty of Medicine, Freiburg, Germany; ^2^ Faculty of Biology, Albert Ludwigs University, Freiburg, Germany; ^3^ BIOSS Centre for Biological Signalling Studies, Albert Ludwigs University Freiburg, Freiburg, Germany; ^4^ Department of Dermatology and Venereology, University Medical Center, Freiburg, Germany; ^5^ Department of Pneumology, University Medical Center, Freiburg, Germany; ^6^ Institut für Molekulare Medizin und Zellforschung, University Medical Center, Freiburg, Germany; ^7^ First Department of Medicine, University of Lübeck, Lübeck, Germany; ^8^ German Cancer Consortium (DKTK), Freiburg, Germany; ^9^ Institute of Experimental Dermatology, University of Lübeck, Lübeck, Germany; ^10^ Department of Immunology, BIOSS Center for Biological Signaling Studies, Faculty of Biology, Albert-Ludwigs-University of Freiburg, Freiburg, Germany; ^11^ Center of Chronic Immunodeficiency CCI, University Clinics and Medical Faculty, Freiburg, Germany; ^12^ Clinical Trials Unit, University Medical Center, Freiburg, Germany; ^13^ The Garvan Institute of Medical Research and The Kinghorn Cancer Centre, Sydney, Australia; ^14^ Department of Pathology, University Medical Center, Faculty of Medicine, Freiburg, Germany; ^15^ German Cancer Research Center (DKFZ), Heidelberg, Germany

**Keywords:** biglycan, melanoma, microenvironment, tissue stiffness, integrin-β1

## Abstract

Novel targeted and immunotherapeutic approaches have revolutionized the treatment of metastatic melanoma. A better understanding of the melanoma-microenvironment, in particular the interaction of cells with extracellular matrix molecules, may help to further improve these new therapeutic strategies.

We observed that the extracellular matrix molecule biglycan (Bgn) was expressed in certain human melanoma cells and primary fibroblasts when evaluated by microarray-based gene expression analysis. Bgn expression in the melanoma tissues correlated with low overall-survival and low progression-free-survival in patients. To understand the functional role of Bgn we used gene-targeted mice lacking functional Bgn. Here we observed that melanoma growth, metastasis-formation and tumor-related death were reduced in *Bgn^−/−^* mice compared to *Bgn^+/+^* mice. *In vitro* invasion of melanoma cells into organotypic-matrices derived from *Bgn^−/−^* fibroblasts was reduced compared to melanoma invasion into *Bgn*-proficient matrices. Tissue stiffness as determined by atomic-force-microscopy was reduced in *Bgn^−/−^* matrices. Isolation of melanoma cells and fibroblasts from the stiffer *Bgn^+/+^* matrices revealed an increase in integrin-β1 expression compared to the *Bgn^−/−^* fibroblast matrices. Overexpression of integrin-β1 in B16-melanoma cells abolished the survival benefit seen in *Bgn^−/−^* mice. Consistent with the studies performed in mice, the abundance of Bgn-expression in human melanoma samples positively correlated with the expression of integrin-β1, which is in agreement with results from the organotypic invasion-assay and the *in vivo* mouse studies.

This study describes a novel role for Bgn-related tissue stiffness in the melanoma-microenvironment via regulation of integrin-β1 expression by melanoma cells in both mice and humans.

## INTRODUCTION

Non-cellular components of the tumor microenvironment, in particular extracellular matrix molecules (ECM), were shown to promote cancer progression via direct effects on tumor cells and indirect effects on fibroblasts, immune cells and angiogenesis [1, 2]. The ECM is formed by tumor stroma and the basement membrane, which differ in their protein, glycoprotein, proteoglycan and polysaccharide composition. A key mechanical characteristic of the ECM is its elasticity. Rigidity of the matrix substrate increases traction forces within the cell and enhances cell migration due to lamellipodia and lamella expansion towards the rigid substrate [3]. Stroma within a tumor microenvironment is typically stiffer than normal stroma [4]. This stiffer tumor stroma is characterized by alterations in collagen architecture, such as linearized and cross-linked collagen, which has been shown to promote tumor cell migration [5]. Mechanistically, enhanced tumor cell motility relies on increased integrin clustering on the cell surface, forming focal adhesions due to altered collagen configuration [2].

The ECM molecule biglycan (Bgn) belongs to the small leucine-rich proteoglycan superfamily [6]. A deficiency in Bgn leads to changes in collagen fibril structure and assembly in bone, tendon and dermis [7]. The expression of Bgn has been studied in several tumor entities. Increased expression of Bgn in gastric cancer [8] and colorectal cancer [9] was shown to be associated with low tumor differentiation, higher frequency of distant metastases and poor prognosis. In addition, Bgn levels were associated with poor prognosis in patients with esophageal squamous cell carcinoma [10] and endometrial cancer [11]. Bgn seems to have roles in both tumor growth and migration, depending on the type of the tumor. In bladder cancer, Bgn is an endogenous inhibitor of bladder cancer cell proliferation [12]. In gastric cancer, Bgn was shown to enhance gastric cancer cell migration and invasion ability, as well as ability of endothelial cells to form tubes [13]. Similarly, knockdown of Bgn in endometrial cancer cells significantly decreased their migration and invasion capacities *in vitro* as well as in a xenograft model [14]. Proteomic analysis of the secretome from tumor cell lines following oncogenic Ras-induced epithelial-mesenchymal transition (EMT) revealed upregulation of Bgn [15], indicating that tumor cells undergoing EMT remodel the ECM via increased Bgn release. Bgn could also play a role in the EMT process, as it has been reported to induce properties in lung fibroblasts similar to those observed during EMT, thereby ultimately increasing cell migration [16].

To date, no functional study on the role of Bgn in melanoma has been reported. We observed that *Bgn^−/−^* mice receiving different melanoma cell lines survived better when compared to *Bgn^+/+^* mice. Functionally, we were able to prove that invasion of melanoma cells into organotypic culture assays was reduced when the fibroblasts in the matrix were derived from *Bgn^−/−^* mice rather than *Bgn^+/+^* mice. This result is due to the decrease the decrease in matrix stiffness in matrices containing *Bgn^−/−^* fibroblasts compared to *Bgn^+/+^* fibroblasts, as measured by atomic force microscopy. Isolation of cells from the stiffer matrices revealed increased integrin-β1 expression compared to those isolated from the less stiff *Bgn^−/−^* fibroblast matrices. In concordance with our mouse and *in vitro* data, we observed a significant survival disadvantage in patients with high Bgn expression in their primary tumor.

## RESULTS

### Bgn expression correlates with tumor invasiveness, overall survival and progression-free survival in human melanoma

**Figure 1:**
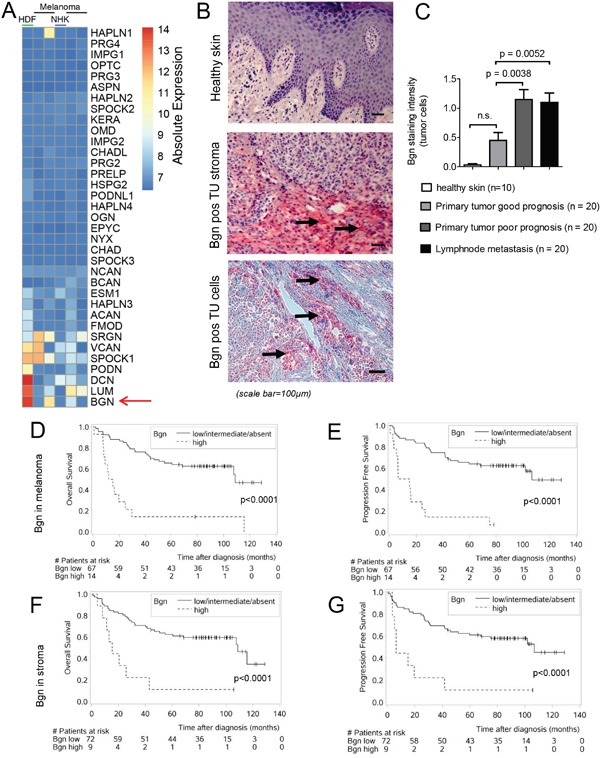
Bgn is highly expressed in melanoma cells and its expression in human melanoma tissue correlates with tumor invasiveness, overall survival and progression-free survival **(A)** Expression of proteoglycans in fibroblast and melanoma cell lines. The heatmap shows the absolute log2 transformed expression of proteoglycans genes as defined in Naba et al. [48] for human dermal fibroblasts (HDF), Normal human keratinocytes (NHK) and 4 melanoma cells lines (detailed in the methods). The expression values have been derived from triplicates. Rows and columns have been hierarchically clustered by a complete linkage method according to their Euclidean distances. **(B)** Representative pictures of human healthy skin, Bgn positive tumor stroma or tumor cells in dermatotropic melanoma metastasis are shown. Immunohistochemical staining for Bgn (red staining, positive cells/areas are indicated by black arrows). **(C)** Quantitative comparison of Bgn staining in normal skin, melanoma primary tumors with good prognosis, melanoma primary tumors with unfavorable prognosis and melanoma lymph node metastases. Patients characteristics are given in Supplementary Table 1. **(D)** Overall survival of the two groups stratified according to Bgn expression levels in the melanoma cells. Characteristics of the patients reported in **(D-G)** are given in Supplementary Table 1. The Bgn level in the melanoma tissue was determined at initial diagnosis in patients with histology-proven malignant melanoma. OS was calculated from the time point of diagnosis until death or last hospital visit. **(E)** Progression free survival of the two groups stratified according to Bgn expression levels in melanoma cells. **(F)** Overall survival of the two groups stratified according to Bgn expression levels in tumor stroma. **(G)** Progression free survival of the two groups stratified according to Bgn expression levels in tumor stroma.

To identify ECM candidates we performed microarray based gene expression analysis of different human melanoma cell lines and human fibroblasts. We observed that the genes DCN (decorin), LUM (lumican) and BGN (Bgn), which are members of the small leucine-rich proteoglycan (SLRP) family [17], were within the highest expressed ECM genes in both melanoma cells and fibroblasts (Figure 1A). Increased expression of Bgn had been reported in different cancer types [8–11], suggesting its role in the tumor microenvironment. However, the mechanism by which Bgn affects tumor growth and metastasis was unclear, which motivated us to study this ECM molecule. To clarify, if the observation that Bgn is upregulated in the cells analyzed by gene expression analysis could be clinically relevant, tissue samples of human primary melanoma and lymph node metastases were analyzed by immunohistochemical staining for Bgn. The results were correlated with clinical outcome. We observed that Bgn was highly expressed in melanoma cells and tumor stroma while it was hardly detectable in normal skin (Figure 1B, 1C, Supplementary Figure 1B). The difference between high levels of Bgn in the primary human fibroblasts and low levels in the healthy skin may be due to multiple other cell types found in the skin, in particular keratinocytes. We found no major expression of Bgn in normal human keratinocytes (NHK) (Figure 1A). Melanoma samples from patients with poor prognosis and those derived from lymph node metastases displayed higher Bgn levels compared to melanoma samples from patients with good prognosis (Figure 1C). A high level of Bgn expression in the tumor cells of human melanoma tissue was associated with reduced overall survival (OS) and progression-free survival (PFS) (Figure 1D, 1E). Furthermore, a high level of Bgn expression in the tumor stroma in these tissue samples was similarly associated with reduced OS and PFS (Figure 1F, 1G). There were only 14 patients with high Bgn expression in the tumor cells. Nevertheless, we still observed a significant difference in OS and PFS when we matched these patients with respect to age and gender to the patients from the Bgn absent/low/intermediate group (Supplementary Figure 1A). The patients with high Bgn in the tumor cells did not necessarily have a high stroma Bgn expression. The overlap between the two groups that had high Bgn levels in both tumor and stroma cells was 3 patients. There were 11 patients that had only high Bgn levels in melanoma cells but not in stroma cells and 6 patients with high Bgn level only in the tumor stroma. Bgn expression was highest in tumor cells, followed by tumor stroma and a lower Bgn expression was also detected in inflammatory cells (Supplementary Figure 1B). In a multivariate analysis, biglycan expression positively correlated with tumor thickness, Clark level and tumor stage (Supplementary Figure 1C-1E). Additionally, Bgn expression was higher in the primary tumors of patients with lymph node metastases at the timepoint of diagnosis (Supplementary Figure 1F).

### Bgn deficiency causes delayed metastatic spread of melanoma cells in mouse models

**Figure 2:**
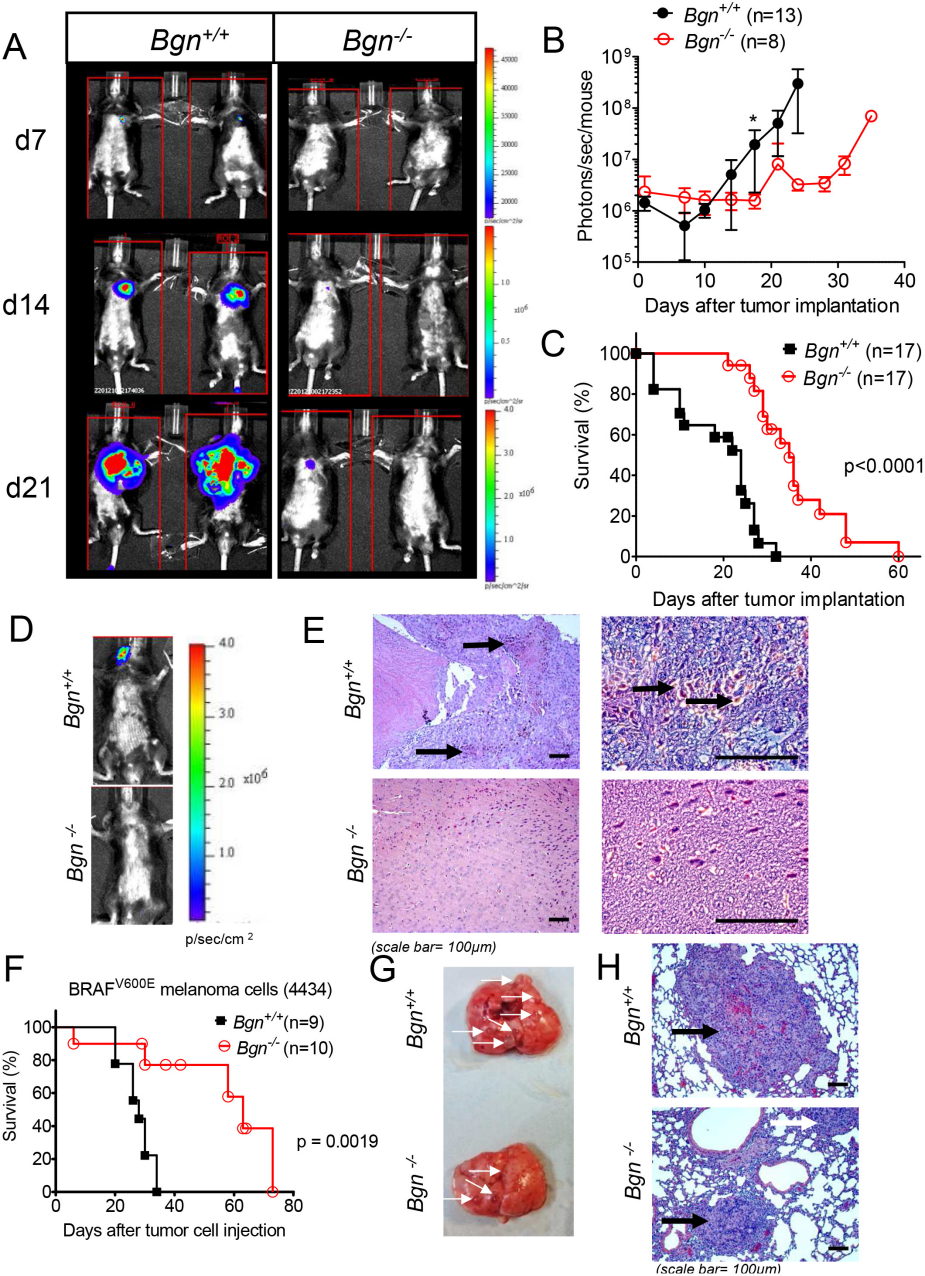
Bgn deficiency of tumor stroma inhibits metastasis formation *in vivo* **(A)** *Bgn^+/+^* and *Bgn^−/−^* mice were injected with 1×10^4^ B16 F10 luciferase transgenic cells intravenously. Distribution of tumor cells was followed by means of bioluminescence imaging on consecutive days. Representative images of tumor bearing mice taken over a period of 21 days. **(B)** The total photon flux over each mouse was quantified in photons/s/mouse as an indicator for the expansion of B16 cells (n=13 or n=8 as indicated). **(C)** Survival was monitored in the groups described under **(A)**. Data are pooled from three independent experiments (n=17 mice/group, *Bgn^+/+^* versus *Bgn^−/−^*: p<0.0001). **(D)** Bioluminescence imaging of *Bgn^+/+^* or *Bgn^−/−^* mice that had received melanoma cells as described under **(A)**. Representative images show BLI signal projection over the head of the mouse. **(E)** The brain of the mice described in **(D)** was isolated on after tumor cell injection and representative images are shown for both groups (H&E stain, black arrows: melanin). **(F)** Survival of *Bgn^+/+^* and *Bgn^−/−^* mice injected intravenously with 2×10^6^ BRAF^V600E^ melanoma (4434) cells (n=9 or n=10 mice/group, *Bgn^+/+^* versus *Bgn^−/−^*: p=0.0019). **(G)** The lung was isolated from the groups described under **(F)** on after tumor cell injection and representative lungs with macroscopically visible metastases are shown for both groups (white arrows point to macroscopically visible metastases). **(H)** The lungs were isolated as described under **(G)** and representative sections of lungs are shown for both groups (H&E stain, black arrows point to melanoma metastases).

To evaluate whether or not Bgn impacts metastatic spread of melanoma cells, we generated luciferase transgenic melanoma cells that contain firefly luciferase as described previously [18]; this transgenic cell line is termed B16-luc^+^. After an intravenous injection, B16-luc^+^ cells were tracked by bioluminescence imaging (BLI) on consecutive days in *Bgn^+/+^* and *Bgn^−/−^* mice. Metastatic spread to the lungs was reduced in *Bgn^−/−^* mice compared to *Bgn^+/+^* mice (Figure 2A). Bioluminescence signal as an indicator for tumor cell expansion was quantified over time and increased more slowly in *Bgn^−/−^* mice compared to *Bgn^+/+^* mice (Figure 2B). Consistent with delayed tumor cell expansion, survival of tumor bearing *Bgn^−/−^* mice was improved compared to *Bgn^+/+^* mice (Figure 2C). In addition to pulmonary metastases, we also observed brain metastases in mice injected intravenously with B16-luc^+^ cells. The metastatic spread to the brain was only observed in *Bgn^+/+^* mice using BLI (Figure 2D) or histology (Figure 2E). To clarify if the less invasive phenotype of melanoma cells in *Bgn^−/−^* mice compared to *Bgn^+/+^* mice was B16 model-dependent, we next transferred a second melanoma cell line (4434), dependent on active BRAF^V600E^ [19], into C57/BL6 mice. Survival was improved in *Bgn^−/−^* mice compared to *Bgn^+/+^* mice (Figure 2F). Consistently, smaller lung metastases were found in *Bgn^−/−^* mice compared to *Bgn^+/+^* mice (Figure 2G, 2H). Bgn was expressed in both mouse melanoma cell lines (B16 and 4434) that we used for *in vivo* experiments (Supplementary Figure 2). These data indicate that the metastatic spread of melanoma cells *in vivo* is more efficient in the presence of Bgn and concurrently indicate that a lack of Bgn significantly delays tumor spread, suggesting a role in metastasis formation.

### Invasion of melanoma cells into organotypic collagen matrices is reduced in the presence of Bgn deficient fibroblasts

**Figure 3:**
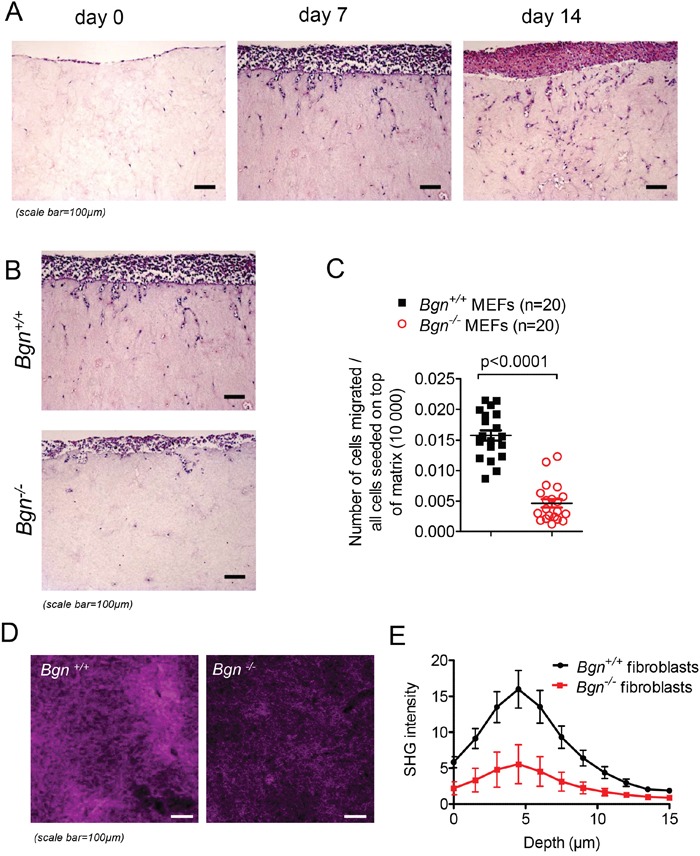
Invasion of melanoma cells is reduced in the absence of Bgn in fibroblasts **(A)** Cells were seeded on the organotypic membranes at 10000 cells/membrane and allowed to adhere for three days. They were allowed to invade into the membranes and histological sections were made on consecutive days. **(B)** Representative images of histological slides of invasive cell migration in *Bgn^+/+^* matrices (top) versus *Bgn^−/−^* matrices (bottom) on day 7 of invasion. **(C)** Quantification of migrated cells per optical field (OF) related to 10 000 cells seeded on the top of the matrix. For each sample, cells in three different optical fields were counted and the average was calculated (n=20/group, *Bgn^+/+^* versus *Bgn^−/−^*: p<0.0001). **(D)** Maximum intensity projection of Second Harmonic Generation (SHG) signal derived from type I collagen in *Bgn^+/+^* and *Bgn^−/−^* organotypic matrices at multiple time points. **(E)** z-stack quantification (0-15 μm) of SHG signal in organotypic *Bgn^+/+^* and *Bgn^−/−^* matrices.

To understand how the lack of Bgn reduced metastasis formation, we next evaluated its impact on cell motility and invasion *in vitro*. To follow invasive migration of tumor cells *in vitro* in an environment resembling *in vivo* conditions as closely as possible, we applied an organotypic invasion assay as previously reported [20]. Mouse embryonic fibroblasts isolated from a *Bgn^−/−^* mouse were immortalized by serial passaging and used in comparison to *Bgn^+/+^* mouse embryonic fibroblasts generated the same way. To generate the matrix, fibroblasts were seeded into collagen, the contraction of which was mediated by the ECM molecules secreted by the fibroblasts. The expression of Bgn in WT fibroblasts was confirmed by Western blot (data not shown). To assess the ability of B16 melanoma cells to migrate in this system, cells were seeded at 10,000 cells per membrane and allowed to adhere for three days. Afterwards, the membranes were placed on metal grids and incubated for a predetermined number of days to allow for tumor cell invasion into the membranes. The histological sections were made on consecutive days and the invasive potential of the cells was evaluated. In a first set of experiments we studied invasion kinetics of melanoma cells into a matrix containing only WT fibroblasts. Over time, an increased invasion of melanoma cells was observed (Figure 3A). To understand whether the modification of matrix characteristics by the lack of Bgn has an impact on tumor cell invasion, we compared the invasion into matrices with fibroblasts derived from *Bgn^−/−^* mice compared to those derived from *Bgn^+/+^* mice. We observed that invasion of melanoma cells into the collagen matrix was reduced when fibroblasts were lacking Bgn as shown for representative matrix sections (Figure 3B). The ratio of invading cells visible on the sections to the total amount of cells seeded on the top of the matrix was lower when fibroblasts were derived from *Bgn^−/−^* mice compared to fibroblasts derived from *Bgn^+/+^* mice (Figure 3C). The same experiment was performed in a different invasion model using isogenic fibroblasts and yielded comparable results (Supplementary Figure 3A-3C). To further assess this phenomenon, we used Second Harmonic Generation (SHG) imaging of collagen matrices following invasion. 3D-SHG imaging revealed a significant reduction of collagen coverage in *Bgn^−/−^* matrices compared to *Bgn^+/+^* matrices (Figure 3D, 3E).

### Bgn deficiency in fibroblasts leads to delayed matrix contraction and decreased matrix stiffness

**Figure 4:**
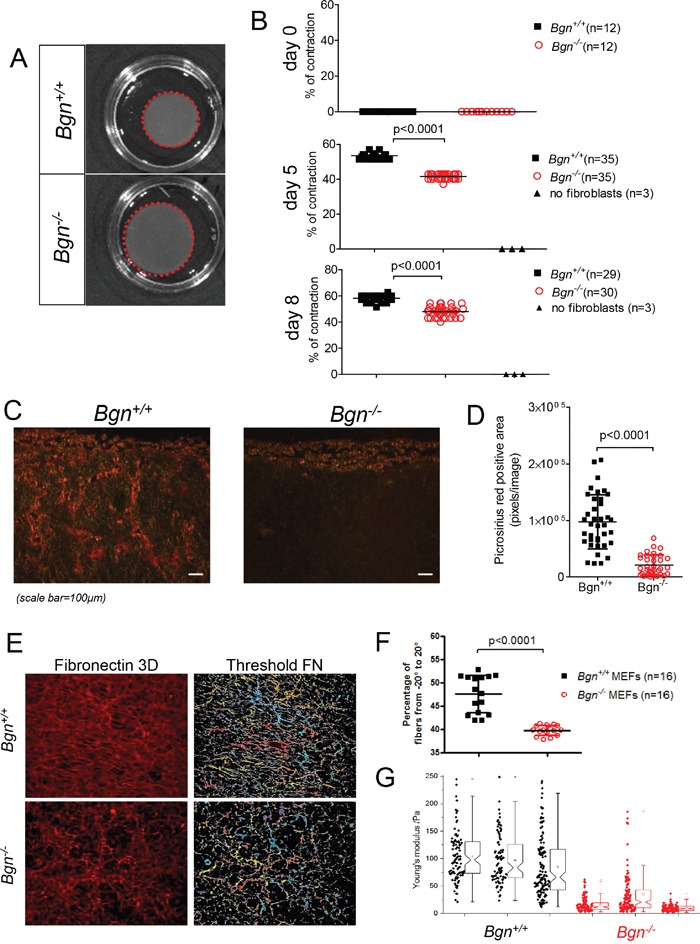
Tissue stiffness is reduced in the absence of Bgn in fibroblasts **(A)** Contraction of collagen matrices containing *Bgn^+/+^* or *Bgn^−/−^* MEFs as indicated for the respective groups. One representative collagen matrix per group is shown. **(B)** Measurement of the diameter of multiple collagen matrices containing *Bgn^+/+^* or *Bgn^−/−^* MEFs on days 5 and 8 after start of contraction. Quantification was determined as the percentage of the initial membrane that contracted, where 0% was the size of a 35 mm Petri dish at the beginning of contraction on day 0. **(C)** Images of picrosirius red staining on sections of collagen matrices containing *Bgn^+/+^* or *Bgn^−/−^* MEFs. Dense, rigid collagen bundles appear orange-red. **(D)** The scatter plot shows quantification of picrosirius red positive areas (in pixels), n ≥ 9 images quantified per condition, values represent mean ± SD. p<0.0001 for *Bgn^+/+^ vs. Bgn^−/−^* membranes. **(E)** Fibroblast derived matrices (FDMs) were fixed and fibronectin fibres stained. **(F)** Statistical analysis of fibronectin fibres orientation using the Mann-Whitney-Test (p<0.0001). Data from 16 samples out of 2 individual experiments were analysed. **(G)** Atomic force microscopy measurements of matrix stiffness of collagen matrices containing *Bgn^+/+^* or *Bgn^−/−^* MEFs. The data were measured on numerous positions on three independent matrices on day 13 of contraction. The Young's moduli of the accepted curves are shown as scatter plots and notched boxplots for all measurements. A two-sample Kolmogorov-Smirnov test indicates a significant difference between the two distributions with a p-value < 0.0001.

A stiffer matrix promotes cell migration via several mechanisms [2, 21]. Since Bgn was shown to interact with collagen I leading to increased cross-linking [7], we hypothesized that the lack of Bgn could impact collagen remodelling in the organotypic invasion assay with consecutive alterations in stiffness. We observed reduced contraction at two time points for organotypic collagen matrices containing *Bgn^−/−^* mouse embryonic fibroblasts compared to those with *Bgn^+/+^* fibroblasts (Figure 4A, 4B). Organotypic collagen matrices lacking fibroblasts did not contract, indicating that fibroblasts and fibroblast-associated molecules are responsible for the ECM remodelling and contraction (Figure 4B). To determine if the lack of Bgn changed collagen fibril density, the matrices were analyzed by picrosirius red staining, allowing for detection of dense collagen bundles as previously described [22]. Matrices containing *Bgn^−/−^* mouse embryonic fibroblasts had a clear reduction in dense collagen bundles compared to matrices with *Bgn^+/+^* fibroblasts (Figure 4C, 4D). Similarly, *Bgn^+/+^* matrices contained more parallel and organized fibronectin fibres than *Bgn^−/−^* matrices, which was quantified as a percentage of fibres with orientation angle from -20° to 20° as previously described [23] (Figure 4E, 4F). To analyze if the reduced contraction of the matrices and the less dense collagen structure correlated with matrix stiffness, indentation-type atomic force microscopy (AFM) was applied to determine the stiffness at numerous positions of the matrices. We observed reduced matrix stiffness when the fibroblasts were derived from *Bgn^−/−^* mice compared to the *Bgn^+/+^* mouse-derived fibroblasts (Figure 4G). These findings indicate that genetic deficiency of Bgn in fibroblasts leads to delayed matrix contraction, less dense collagen bundles and decreased matrix stiffness.

### Integrin-β1 expression correlates with tissue stiffness, is decreased in *Bgn^−/−^* matrices and correlates with Bgn expression in humans

**Figure 5:**
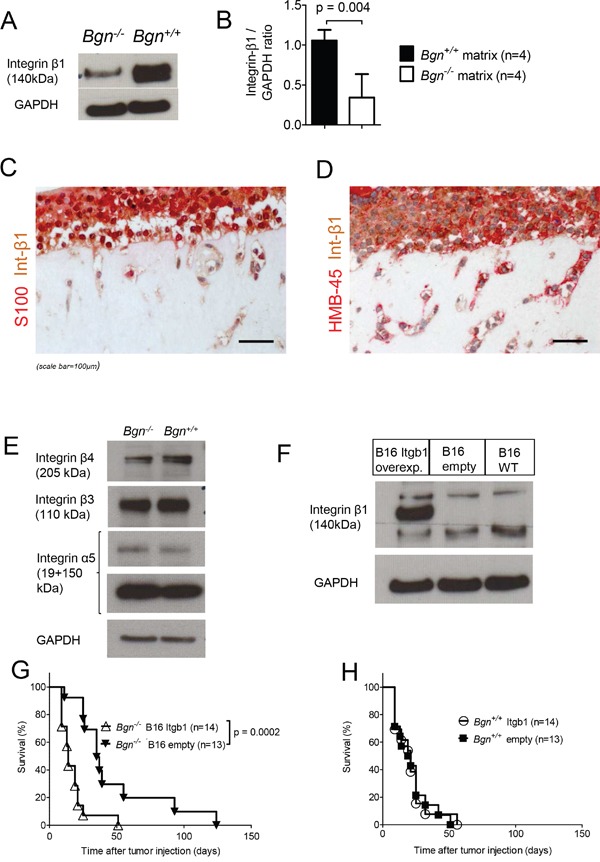
Stiffness mediated cell invasion is integrin-β1 dependent **(A)** A representative western blot analysis for integrin-β1 in cells isolated from *Bgn^+/+^* matrices vs. *Bgn^−/−^* matrices is shown. The cells had been allowed to invade the matrix for 7 days. Membranes were digested with collagenase 1, cells were isolated and western blot was performed. GAPDH was used as control. **(B)** The bar diagram shows the quantification of integrin-β1 in cells isolated from *Bgn^+/+^* vs. *Bgn^−/−^* collagen matrices, normalized to GAPDH. Data are pooled from four independent experiments. p=0.004 for *Bgn^+/+^ vs. Bgn^−/−^* membranes. **(C)** Double immunohistochemistry staining for S100 and integrin-β1 of matrices containing melanoma cells is shown. **(D)** Double immunohistochemistry staining for HMB-45 and integrin-β1 of matrices containing melanoma cells is shown. **(E)** Western blot analysis of different integrins in cells isolated from *Bgn^+/+^* vs. *Bgn^−/−^* collagen matrices. Representative results are shown. GAPDH was used as control. **(F)** Expression of integrin-β1 in B16 WT cells or cells containing the integrin-β1 overexpression vector as shown by western blot analysis. **(G)** Survival of *Bgn^−/−^* mice injected with either 1×10^4^ B16 integrin-β1 overexpressing cells or with 1×10^4^ B16 cells bearing the empty vector (n=14 or n=13 mice/group, integrin-β1 overexpressing cells versus cells bearing the empty vector: p=0.0002). **(H)** Survival of *Bgn^+/+^* mice injected with 1×10^4^ B16 integrin-β1 overexpressing cells or with 1×10^4^ B16 cells bearing the empty vector (n=14 or n=13 mice/group, integrin-β1 overexpressing cells versus cells bearing the empty vector: not significant).

To analyze the mechanism leading to enhanced cell migration in the stiffer matrices, cells were isolated from *Bgn^−/−^* or *Bgn^+/+^* fibroblast-derived matrices. Protein analysis for multiple integrins revealed reduced integrin-β1 expression when cells were isolated from *Bgn^−/−^* matrices compared to *Bgn^+/+^* matrices (Figure 5A, 5B). Immunohistochemical analysis showed that integrin-β1 expression was found in both S100^+^/HMGB-1^+^ melanoma cells and fibroblasts within the matrices (Figure 5C, 5D). Since melanoma cells were several fold higher in numbers than fibroblasts in the matrices, the levels of integrin-β1determined by western blot represent mainly the expression of this molecule in melanoma cells. The increased expression upon contact with WT matrices was most prominent for integrin-β1 but also to a minor degree visible for integrin-β4 (Figure 5E). We could not detect higher integrin-β1 expression in the melanoma cells after exposure to soluble Bgn or conditioned media from the *Bgn^+/+^* MEFs (Supplementary Figure 4A-4D), indicating that the effect was connected to higher tissue stiffness, rather than a direct effect of the soluble Bgn on melanoma cells.

To understand if the low integrin-β1 expression/low invasiveness phenotype of melanoma cells derived from a *Bgn^−/−^* matrix could be reversed, we overexpressed integrin-β1 in B16 melanoma cells. Overexpression of integrin-β1 was effective (Figure 5F) and the injection of the integrin-β1 overexpressing melanoma cells (B16) abolished the survival benefit we had previously seen in *Bgn^−/−^* mice (Figure 5G). Survival of melanoma bearing mice was altogether slightly better than in previous experiments using unmodified melanoma cells. This could be due to the genetic modification of the cells with empty vector or integrin- β1 vector. Consistent with this concept, we observed a slower growth of the gene modified B16 cells in the cell culture. Consistent with the concept that integrin-β1 overexpression reversed the Bgn-related survival benefit, no enhanced tumor-related mortality was seen when integrin-β1 overexpressing melanoma cells were compared to empty-vector melanoma cells in *Bgn^+/+^* mice (Figure 5H). These findings indicate a functional connection between the lack of Bgn leading to low integrin-β1 expression and reduced melanoma invasiveness seen *in vivo*.

**Figure 6:**
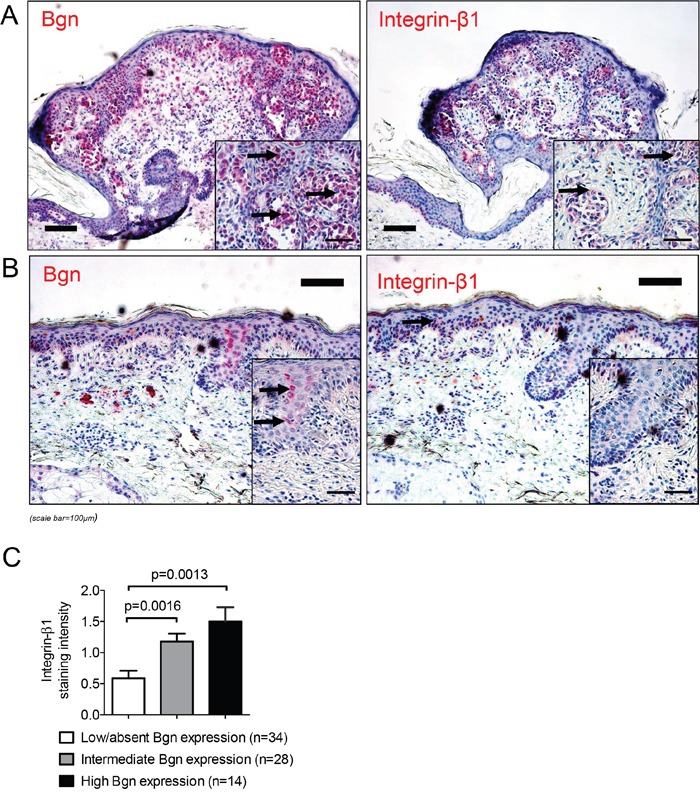
The levels of Bgn and integrin-β1 in melanoma tissue correlate **(A)** Immunohistochemical staining of Bgn (left image) and integrin-β1 (right image) in a representative human melanoma sample with high Bgn and integrin-β1 expression levels (black arrows point to red staining of Bgn and integrin-β1). **(B)** Low staining intensity of integrin-β1 in a patient sample with low biglycan staining intensity (black arrows point to red staining of Bgn and integrin-β1). **(C)** Quantification of integrin-β1 in the primary tumors of patients stratified according to low/absent, intermediate and high Bgn expression. Patients characteristics are given in Supplementary Table 1.

To evaluate if these findings in the mouse model could be relevant for human situation, we next analyzed integrin-β1 expression in human tumor tissues and found it to be directly correlating with the level of Bgn, as shown for a representative patient with high Bgn/integrin-β1 expression (Figure 6A) compared to a different patient with low Bgn/integrin-β1 expression (Figure 6B). The level of Bgn expression correlated with integrin-β1 expression (Figure 6C), consistent with the functional connection of Bgn and Integrin-β1 that we had seen in the mouse model. Consistent with the finding that there was hardly any Bgn detectable in the normal skin, we could also find only a very low integrin-β1 expression in the normal skin (Supplementary Figure 4E). This supports the concept that the expression levels of both molecules correlate.

## DISCUSSION

The tumor microenvironment is a dynamic niche created by the tumor and tumor-derived interactions with surrounding cells and the ECM. The tumor cells modify this niche to promote growth, progression and metastasis. Tissue stiffness based on the organization of collagen, the major component of the ECM, directly correlates with tumor invasiveness and activation of the regulatory circuitry including amongst others ERK, Rho and ROCK activity, ultimately promoting tumor cell motility [2, 24]. Based on the central role of collagen in the tumor microenvironment, we analyzed the ECM component Bgn, which has been shown to interact with different collagen subtypes [25–28] and could therefore potentially influence tissue stiffness. We were able to observe reduced melanoma growth *in vivo* in mice lacking Bgn and less invasion *in vitro* when Bgn deficient fibroblasts were used in the respective organotypic matrix. Functionally, we linked this decreased invasiveness into Bgn deficient matrices to reduced matrix stiffness and diminished expression of integrin-β1 within the matrix. Complementary to the findings in the mouse model, we observed that patients with high Bgn expression levels in the melanoma tissue experienced a poor overall- and progression-free-survival. Consistent with the functional connection between Bgn and integrin-β1 we had described in the mouse model we found a correlation between Bgn and integrin-β1 expression in human melanoma tissues.

Our findings on the role of Bgn in matrix organisation are consistent with previous reports on the interaction of Bgn with collagen. Bgn has been shown to interact with several collagen subtypes including collagen I [25], collagen II [26], collagen VI [27] and collagen IX [28] and its lack results in collagen fibrils being more loosely organized [7]. Consistent with the published literature, we found a loosened organization of collagen fibrils in matrices containing Bgn deficient fibroblasts, as quantified by Picrosirius staining. Similarly, we measured lower tissue stiffness in Bgn deficient matrices by atomic force microscopy. Our finding that stiffer tissue correlates with increased invasiveness of melanoma cells is consistent with results from other reports examining mammary gland duct epithelial cells [2], keratinocytes [24] and skin carcinoma cells [22]. Our data extend the findings of these authors because melanoma cells are of neuroectodermal origin, in contrast to epithelial cells in the skin and mammary gland duct derived from the external ectoderm. They therefore represent an ontogenetically different type of cancer cells. We observed that the reduced stiffness of the Bgn deficient matrix leads to a decreased cell migration. A possible explanation of this effect is a reduction in integrin-β1 expression that we show in our *in vitro* system, as well as in the human samples. This is consistent with reports from others showing that different types of integrins are regulated in tumor cells in response to ECM matrix stiffness and composition [5]. Increased stiffness has been shown to lead to enhanced expression of integrin-β1 in MCF-10A mammary epithelial acini, and to increased levels of integrin-α6 and integrin-β4 in mammary gland ducts [2]. In addition, it was shown that exogenous force can activate integrins [29, 30] and promote adhesion assembly [31]. This then enhances focal adhesion formation, thereby promoting tumor cell motility. Besides their functional role in cell motility, integrins are also transmembrane ECM receptors that transmit information due to mechanical stress from outside into the cell [2]. We did not observe an increase in integrin-β1 expression after exposing the melanoma cells to soluble Bgn or conditioned media of Bgn*^+/+^* MEFs. This supports the hypothesis that Bgn enhances integrin-β1 expression and cell migration via increased tissue stiffness. When integrin-β1 was overexpressed, the protective phenotype of Bgn deficient mice was antagonized. We were able to link decreased tissue stiffness and lower melanoma invasiveness to reduced integrin-β1 expression. Consistent with the mouse data, levels of Bgn correlated with the integrin-β1 levels in human samples, providing an indirect evidence for a functional connection between Bgn and integrin-β1 in humans as well.

A limitation of our study is that, while patient samples were taken from the primary tumor and lymph node samples, a murine model for distant metastasis was chosen, thereby describing the process of extravasation and metastatic growth. In this study we focused only on the role of Bgn in the tumor microenvironment and the extracellular matrix. Nevertheless, the high Bgn expression in the tumor cells that we observed suggests that Bgn may also have a cell-intrinsic role in tumor cells.

Our findings on Bgn in the melanoma model may not be applicable to all other tumor entities, as previous reports indicate that Bgn can have different effects on tumor growth, depending on the type of tumor. Other investigators had shown in an approach different from our studies that incubation of pancreatic cancer cells with Bgn led to reduced growth, specifically, the cell cycle was halted by inducing a G1 arrest. This was accompanied by p27 up-regulation and down-regulation of cyclin A, as well as by a decrease in RAS and ERK activation [32]. In contrast to these results, multivariate analyses of pancreatic carcinoma patient samples in a later report identified Bgn as a negative prognostic factor [33]. Upregulated Bgn expression in human gastric [8] and colorectal cancer [9] was associated with poor survival, higher metastatic potential, higher recurrence rate and poorer differentiation. Bgn expression promoted gastric cancer cell migration and invasion and metastatic ability, *in vitro* as well as *in vivo*. This was achieved by an increase in phosphorylation of FAK and paxilin in the tumor cells [13]. Fibroblasts and melanoma cells displayed higher proliferative and migratory capacity when Bgn expression was downregulated in a HER-2/neu-mediated tumor model [34]. Apart from its role in tumor cells, Bgn was shown to be expressed in human and mouse tumor endothelial cells and to play an important role for their migration and tube formation [13, 35, 36].

In summary, our data clarify the role of Bgn in the melanoma microenvironment. We show that high Bgn expression levels promote a more dense collagen architecture, leading to increased tissue stiffness. This increased tissue stiffness leads to higher integrin-β1 expression on melanoma cells, which promotes their invasiveness.

## MATERIALS AND METHODS

### Patients and tissue specimens

The study included formalin-fixed and paraffin-embedded (FFPE) tissue specimens of primary tumors and lymph node metastases from 81 patients that underwent surgery for the treatment of melanoma. The study had been approved by the local ethics committee (protocol no.: 569/15; Ethic committee, Albert-Ludwigs-University, Freiburg, Germany). Melanoma patients treated within a time period of 10 years (2002-2012) at the Dept. of Dermatology, University Hospital Freiburg, Germany, from whom a biopsy was available, were analyzed. Patients were divided according to their prognosis into the group with a poor prognosis (melanoma-related death within the period), versus the group with a good prognosis (alive within the period), according to previous stratification approaches [37, 38]. More detailed patient characteristics are listed in Supplementary Table 1.

The slides of the melanoma biopsy samples were scored by 2 independent scorers in the Department of Dermatology, who were not aware of each other's results. In case of different results, the mean percentage was taken and the scorers met and discussed the results of the discordant slides.

### Mice

Mice were purchased from the local animal facility of the University Medical Center Freiburg, Germany and bred under special pathogen-free conditions in the Mouse Experimental Unit of the animal facility. C3.129S4(B6)-Bgntm1Mfy *Bgn^−/−^* mice were purchased from Mutant Mouse Regional Resource Centers, Missouri, USA and backcrossed into the C57BL/6 background for more than 6 generations. The genotype of gene-targeted mice was confirmed by polymerase chain reaction (PCR). Mice were used for *in vivo* experiments at between 6 and 12 weeks of age and 20-25 g of weight. The male and female sex were used equally in both *Bgn^+/+^* and *Bgn^−/−^* group. All animal protocols had been approved by the University Committee on the Use and Care of Laboratory Animals at the Albert-Ludwigs University Freiburg, Germany (Protocol approval number: G-13/116).

### Antibodies

Anti-biglycan antibody (Abcam, ab49701 [39]), anti-integrin-β1 Antibody (clone: EP1041Y) (Abcam, ab52971 [40]), anti-Integrin-α5 (Abcam, ab150361) [41], integrin-β4 (Santa Cruz Biotechnology, INC, sc 9090 [42]), anti-Integrin-β3 (clone: D7×3P, Cell Signaling Technology, 13166 [43]). Dilutions of the antibodies are listed in the Supplementary methods.

### Cell lines

B16 F10 murine melanoma cell line was provided by Prof. H. Pircher, Freiburg, Germany. BRAF-mutant 4434 mouse melanoma cell lines were established from C57BL/6_BRAF +/LSL-BRAFV600E; Tyr::CreERT2+/o mice [19] and were provided by Dr. Richard Marais, Manchester, UK. The *Bgn*^+/+^ mouse embryonic fibroblasts (MEFs), obtained from 13.5 day old C57BL/6 mouse embryos and immortalized by serial passaging, were provided by Dr. Arnim Weber, Freiburg, Germany. The *Bgn*^−/−^ MEFs were obtained from 13.5 day old C3.129S4(B6)-Bgntm1Mfy mouse embryos and immortalized by serial passaging. For further series of experiments, *Bgn*^+/+^ and *Bgn*^−/−^ MEFs on a C3H genetic background were obtained as described in Supplementary methods. Primary human dermal fibroblasts (HDF) were isolated from the human foreskin as previously published [44]. The human melanoma cell lines (MaMel) were isolated and cultured as described [45]. Cells were kept under a humidified environment with 5% CO2 and 37°C. Total RNA for microarray was extracted from cells using the Universal RNA Purification Kit (GeneMatrix) kit from Roboklon (Germany) according to the manufacturer's instructions. RNA quality and integrity was verified using the Agilent 2100 Bioanalyzer system (Agilent Technologies, Palo Alto, CA, USA), and its content quantified by NanoDrop ND-1000 (Thermo Fisher Scientific, Wilmington, USA). The cell lines were authenticated by Eurofins Medigenomix GmbH, Ebersberg, Germany.

### Generation of luciferase-expressing B16 F10 mouse melanoma cells

To investigate metastasis formation, luciferase expressing B16 F10 cells (B16 luc^+^) were used. To generate these cells a lentiviral transduction for stable lines was performed as previously described [18].

### Generation of integrin-β1 overexpressing B16 F10 mouse melanoma cells

The expression vector pCMV6-AC-GFP integrin-β1 (Origene) or the respective empty vector (BIOSS toolbox repository) were used. The circular plasmid DNA containing the integrin-β1 gene, a C-terminal tGFP tag, and a gene for neomycin resistance was transformed by heat shock into Subcloning Efficiency DH5alpha Competent Cells (Invitrogen).

All plasmids were extracted from bacterial clones using the Qiagen Maxi-prep Kit. All plasmids were linearized using *Sca*I (New England Biolabs) followed by ethanol precipitation using standard protocols. Precipitated DNA was dissolved in water and transfected into B16 F10 mouse melanoma cells using PolyFect (Qiagen) according to the protocol supplied by the manufacturer. Transfected cells were selected using neomycin and the transfection efficiency was confirmed by flow cytometry analysis for GFP (data not shown) and by Western blot.

### Mouse melanoma model

*Bgn^+/+^* and *Bgn^−/−^* mice were both injected with 1×10^4^ B16 F10 luc^+^ melanoma cells in the tail vein. Afterwards, their survival was monitored and BLI was performed as described [46]. In another experiment, the mice were sacrificed on day 18 and the brain tumors were further examined. Integrin-β1 overexpressing B16 F10 cells/control B16 F10 cells were injected in equal numbers and survival was monitored. For the second melanoma model, 2×10^6^ 4434 melanoma cells were injected intravenously in the tail vein and either survival was monitored or the mice were sacrificed on day 33 and the lung tumors were further examined.

### Bioluminescence imaging

For *in vivo* bioluminescence imaging (BLI), luciferin [D-Luciferin 1-(4,5-dimethoxy-2-nitrophenyl)-ethyl-ester; Biosynth] was injected intraperitoneally at a concentration of 150 μg/g body weight [46]. After 10 minutes, mice were imaged using an IVIS100 CCD imaging system (Xenogen) with an exposure time of 5 minutes. The signal from luciferase transgenic cells was quantified in photons per second per mouse. Acquisition, analysis and visualisation of bioluminescence imaging data was done with Living Image v2.1® (Calipers).

### Western blot

Proteins were separated by SDS polyacrylamide gel electrophoresis. Protein was used in concentrations between 3-10 μg supplemented with 6.25 μl NuPageLDS sample buffer (Invitrogen) and 2.5 μl Reducing agent (Invitrogen) and distilled water was added to a total volume of 18 μl. This reducing sample mix was incubated for 10 minutes at 75°C. Afterwards the samples (18 μl) and pre-stained protein ladder (5-7 μl) were applied to a 4-12% gradient Bis-TRIS gel. Gels were run submerged in 1x NuPage running buffer (+ 500 μl Antioxidant per chamber) in the Invitrogen system at 180 V for 45-60 min. Images were cropped.

### Organotypic invasion assay

The organotypic invasion assay is a method using a 3-dimensional matrix to observe invasive cell migration [20]. The matrix consists of collagen I, and immortalized mouse embryonic fibroblasts. The collagen I originates from adolescent rat tails, having been isolated via an acid extraction which preserves the poly-peptide ends and enables cross-linking. This matrix resembles collagen *in vivo* and provides an ideal environment to follow the invasive properties of cells allowed to migrate through this matrix. For a detailed description see Supplementary methods.

### Second harmonic generation imaging of collagen I

Collagen second harmonic signal was acquired using a 20X 0.95 NA water objective mounted on an inverted Nikon TE-2000 microscope body. The excitation source was a Ti:Sapphire femto-second laser cavity (Coherent Chameleon Ultra II), coupled into a LaVision Biotec Trim-scope scan-head. 880 nm excitation wavelength was used to collect SHG signal (440 ± 20 nm) from collagen I. For each sample, 3 representative regions of interest of 512 μm X 512 μm were imaged over a 3D z-stack (15 μm depth). The percentage area covered by collagen was derived from the intensity of the second harmonic generation (SHG) signal.

### Immunohistochemistry

FFPE tissue specimens (included in tissue microarrays) were cut at 3μm thickness, deparaffinized and subjected to antigen retrieval in pH 6.1 citrate-buffer. Subsequently, immunohistochemical staining was done by incubation with the primary anti-Bgn antibody (Abcam, ab49701) or anti-integrin-β1 antibody (clone: EP1041Y, Abcam, ab52971). Bgn and integrin-β1 expression was evaluated in each of 3 separate high power fields (HPF) with a HPF being a 40x magnification. Slides were scored for Bgn and integrin-β1 intensity as follows: score 0 = negative or weak positive expression, score 1 = moderately positive expression and score 2 = strongly positive expression.

### Gene expression analysis by microarray

Total RNA from the HDF and MaMel cell lines were isolated, labeled and hybridized in triplicates to Illumina HumanHT12-v4 BeadChips (Illumina, San Diego, CA, USA) according to the manufacturer's protocol. Raw microarray data were chip-wise processed using the Bioconductor R package beadarray [47] and subsequently quantile normalized together. Illumina Probes were mapped to reannotated Entrez IDs using the Illumina Human v4 annotation data (Version 1.26) from Bioconductor. If several probes mapped to the same Entrez ID, the one having the largest interquartile range was retained.

### Indentation-type atomic force microscopy

Indentation-type atomic force microscopy (AFM) is well suited for measuring the elasticity of soft collagen-containing matrices [21]. Both wildtype- and Bgn−/− MEF contracted collagen matrices were measured on the same day after 13 days of contraction. More details are in the Supplementary methods.

### Statistical analysis

Statistical analyses were performed with GraphPad Prism Version 4/5 software. Data are reported as mean ± SEM or SD. Kaplan-Meier survival curves were analyzed by log-rank tests (Mantel-Cox test). The Mann-Whitney test was used for comparison of Bgn staining intensity and fibronectin fibre orientation a two-sample Kolmogorov-Smirnov test was used for the evaluation of the atomic force microscopy measurements. Comparisons among groups in other experiments were performed with a 2-tailed unpaired Student *t* test. *P* values < .05 were considered to be statistically significant.

## SUPPLEMENTARY FIGURES AND TABLE



## Abbreviations

Bgn: biglycan
ECM: extracellular matrix
FDM: fibroblast derived matrix
BLI: bioluminescence imaging
MEF: mouse embryonic fibroblasts
NHK: normal human keratinocytes
HDF: human dermal fibroblasts
AFM: atomic force microscopy
SHG: second harmonic generation
IHC: immunohistochemistry
OS: overall survival
PFS: progression free survival
H&E: hematoxylin and eosin stain
