# Supplementary material for: Biglycan expression in the melanoma microenvironment promotes invasiveness via increased tissue stiffness inducing integrin-β1 expression
Source: Oncotarget. 2017 Apr 17;8(26):42901–16. doi: 10.18632/oncotarget.17160 (PMC5522114; doi:10.18632/oncotarget.17160)
Supplement: Supplementary file 1 [file oncotarget-08-42901-s001.pdf]

Biglycan expression in the melanoma microenvironment promotes invasiveness via increased tissue stiffness inducing integrin-β1 expression

SUPPLEMENTARY MATERIALS

SUPPLEMENTARY DATA

Dilution of antibodies

Anti-biglycan (Abcam, ab49701) 1:150 (Immunohistochemistry).

Anti-integrin-β1 Antibody (clone: EP1041Y) (Abcam, ab52971) 1:1000 (Western blot), 1:500 (Immunohistochemistry).

Anti-integrin-α5 (Abcam, ab150361) 1:2000 (Western blot).

Anti-integrin-β4 (Santa Cruz Biotechnology, INC)1:1000 (Western blot).

Anti-integrin-β3 (clone: D7X3P, Cell Signaling Technology, 13166) 1:1000 (Western blot).

Organotypic invasion assay

Collagen I was isolated from rat tails using acid extraction. Mouse embryonic fibroblasts (immortalized) directed the polymerization of collagen thus synthesizing the membranes for cell invasion. Fibroblasts were provided collagen 1 and placed in 35 mm Petri dishes, the consequent polymerization of the collagen formed membranes of a size smaller than 15 mm. The membranes were then incubated with a suspension of $1x10^4$ melanoma cells for three days, initially, to be later placed on metal grid platforms. An air/liquid gradient drove invasive cell migration in direction of the media. After a 7 day migration period, membranes were fixed with paraformaldehyde and embedded in paraffin blocks. Thereafter the paraffin-embedded membranes were sectioned and stained with hematoxylin/eosin. Cell migration was quantified as the median of cells invading the membrane. This was calculated by taking the median of three microscopic fields at 10x magnification. For protein expression analysis, cells were isolated from the membranes after 7 days of migration using collagenase 1. The method was done as previously described (1).

Generation of MEFs on C3H genetic background

For a further series of organotypic invasion assay experiments, $Bgn^{+/+}$ MEFs on C3H genetic background were obtained from Invivogen. These cells were isolated from 13.5 day old C3H WT mouse embryos and immortalized by stable transfection of an SV40 large antigen-expressing plasmid. To be able to compare the two cell lines, C3H derived $Bgn^{-/-}$ MEFs were immortalized using the same SV40 large antigen-expressing plasmid that was used for the other cells.

Picrosirius red staining

Paraffin-embedded organotypic cultures after 7 days of cell migration were sectioned and stained with Weigert's Hematoxylin for 8 minutes and counterstained with 0.1% Picrosirius Red (Direct Red 80; Sigma Aldrich) for 90 minutes as described previously (2). Images were taken using Axioplan2 fluorescence microscope (Zeiss) equipped with an analyzer and polarizer and Axiocam camera. All the images were taken using identical settings and exposure time. Collagen fibril density was quantified on images taken with orthogonally oriented polarized light using image J software. After setting a minimal threshold, the area (in pixel) of the brightness of the threshold light was calculated from a minimum of 9 images per condition.

Indentation-type atomic force microscopy

Indentation-type atomic force microscopy (AFM) is well suited for measuring the elasticity of soft collagen-containing matrices (3). Both WT- and Bgn-KO-MEFs contracted collagen matrices were measured on the same day after 13 days of contraction. Each sample was prepared directly before the experiment. Since the matrices were very soft and also fragile, it was crucial not to apply any excess force on the regions of interest during sample preparation. The individual matrices were gently transferred out of the 35mm Petri dish by completely filling the dish with buffer solution, closing the lid and flipping it upside down. As a consequence, the matrix floated into the lid. The 35mm dish was lifted away from the lid and replaced by a 100mm dish (without lid). After flipping this assembly again and removing the lid the collagen matrix was inside the larger dish and the buffer solution was dispersed. Next, a circular piece was punched out of the center using a 8mm tissue punch. A coverslip (22mm x 22mm) was glued on top of this piece using cyanoacrylate. The glass was used to lift this assembly with tweezers and to glue it into a 60mm dish which was

filled immediately with PBS and used for the elasticity measurements.

Indentation experiments were performed on a JPK NanoWizard 3 BioScience AFM system equipped with a CellHesion 200 module, motorized x-y-precision-stage and TopView optics for sample alignment (JPK Instruments). Spherical indenters were prepared by gluing glass beads (PGB-007, Kisker Biotech) to soft tipless AFM cantilevers (MikroMasch CSC38/tipless/No Al, nominal spring constant 0.03 Nm^{-1}, NanoandMore) using ultraviolet curing glue (Norland Optical Adhesive 61, Norland Products). For the gluing procedure the AFM was mounted on an inverted optical microscope (Nikon Eclipse Ti-E with Intensilight as UV source for glue curing, Nikon Instruments). The bead radius was determined using the optical microscope. All measurements were performed with the same cantilever ($r_{indenter}$=32µm). For force calibration, the nominal spring constant was used and the sensitivity was determined before the experiment series on bare glass in PBS buffer.

The indentation experiments were carried out in 60mm Petri dishes filled with PBS. The length of the force-distance curves was 25µm, the force setpoint was 1.5nN and the extend/retract speed was 5µm/s. On a central region of each sample between 100 and 150 force–distance curves were recorded on different points that were arranged like a grid and separated by at least 50µm. For both phenotypes three different matrices were measured and the respective curves were pooled for data analysis. The Young's moduli were determined by fitting the individual curves to a Hertz model using the JPK data processing software. Curves were rejected if they clearly deviated from a simple Hertzian-type indentation or if the fit residuals were significantly higher than for a typical indentation curve from the respective condition. The data are from the following number of curves: WT-MEFs (n=3) 349 curves were measured, 340 accepted, 9 discarded (2.6%). Bgn-KO-MEFs, (n=3) 415 curves measured, 391 accepted, 24 discarded (5.8%). The Young's moduli of the accepted curves are shown as scatter plots and notched boxplots in Figure 3. A two-sample Kolmogorov-Smirnov test indicates a significant difference between the two distributions with a p-value < 0.0001. OriginPro (Originlab) was used for data plotting and statistical testing.

Fibronectin fiber orientation

Bgn$^{+/+}$ and *Bgn*$^{-/-}$ MEFs were plated on 15 µ-ibidi slides. After 5 days the samples were fixed, permeabilized and stained with anti-Fibronectin antibody (Sigma) and Hoechst33342. 15 µm Z-stacks were imaged and a maximal projection was analysed using MetaMorph. Tresholded fibers with less than 5 pixels were excluded. Percentages of distributed angles of fibers from -20° to 20° were combined as a measure for parallel/organised fibers as previously described (4). For comparison of the two groups the Mann-Whitney-Test was performed.

REFERENCES

1. Timpson P, McGhee EJ, Erami Z, Nobis M, Quinn JA, Edward M, Anderson KI. Organotypic collagen I assay: a malleable platform to assess cell behaviour in a 3-dimensional context. J Vis Exp. 2011;e3089.
2. Mittapalli VR, Madl J, Löffek S, Kiritsi D, Kern JS, Römer W, Nyström A, Bruckner-Tuderman L. Injury-Driven Stiffening of the Dermis Expedites Skin Carcinoma Progression. Cancer Res. 2016; 76:940-51.
3. Calvo F, Ege N, Grande-Garcia A, Hooper S, Jenkins RP, Chaudhry SI, Harrington K, Williamson P, Moeendarbary E, Charras G, Sahai E. Mechanotransduction and YAP-dependent matrix remodelling is required for the generation and maintenance of cancer-associated fibroblasts. Nat Cell Biol. 2013; 15:637-46.
4. Goetz JG, Minguet S, Navarro-Lérida I, Lazcano JJ, Samaniego R, Calvo E, Tello M, Osteso-Ibáñez T, Pellinen T, Echarri A, Cerezo A, Klein-Szanto AJ, Garcia R, et al. Biomechanical remodeling of the microenvironment by stromal caveolin-1 favors tumor invasion and metastasis. Cell. 2011; 146:148-63.

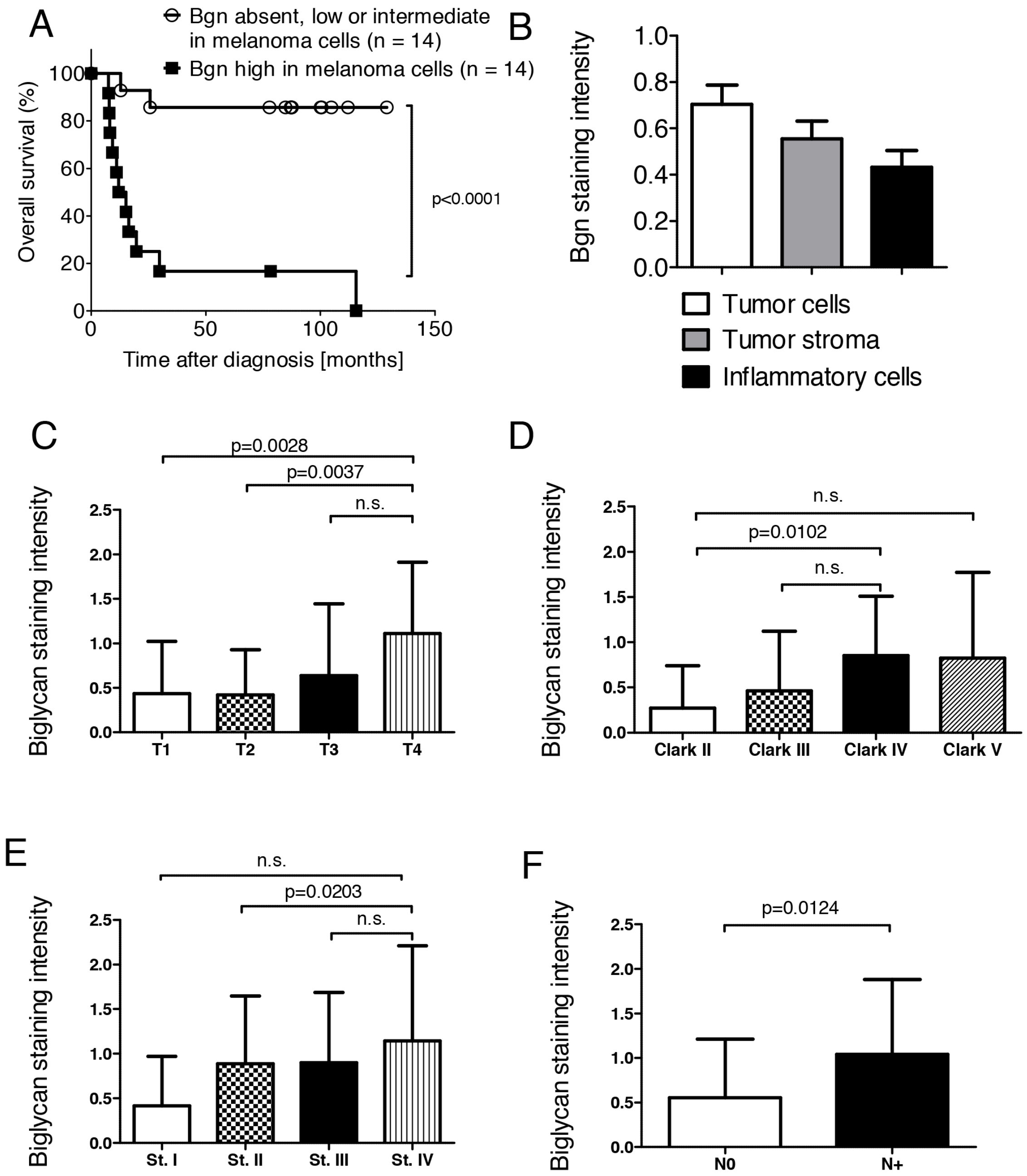

Supplementary Figure 1: Bgn expression correlates with poor survival, tumor thickness, Clark level, tumor stage and the presence of lymph node metastases in human melanoma. **(A)** Patients from the Bgn high expression group were matched with comparable patients from absent/low/intermediate group based on age and gender and a survival analysis was performed (n=14/group, OS high versus absent/low/intermediate: $p<0.0001$). **(B)** Bgn expression levels in tumor cells, stroma cells and immune cells were determined by IHC. **(C)** Quantitative comparison of Bgn staining in primary tumor with groups according to tumor thickness. **(D)** Quantitative comparison of Bgn staining in primary tumor with groups according to Clark level. **(E)** Bgn staining intensity in primary tumors of different tumor stages at the time point of diagnosis. **(F)** Bgn staining intensity in primary tumors of patients with or without lymph node metastases at the time point of diagnosis.

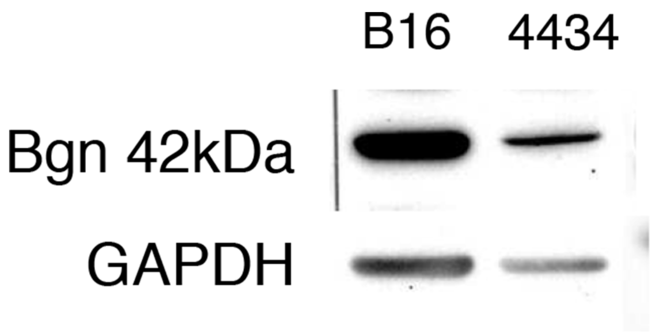

Supplementary Figure 2: Bgn expression in B16 and 4434 cells. A representative western blot analysis of Bgn expression in the B16 and 4434 mouse melanoma cell lines. GAPDH was used as control.

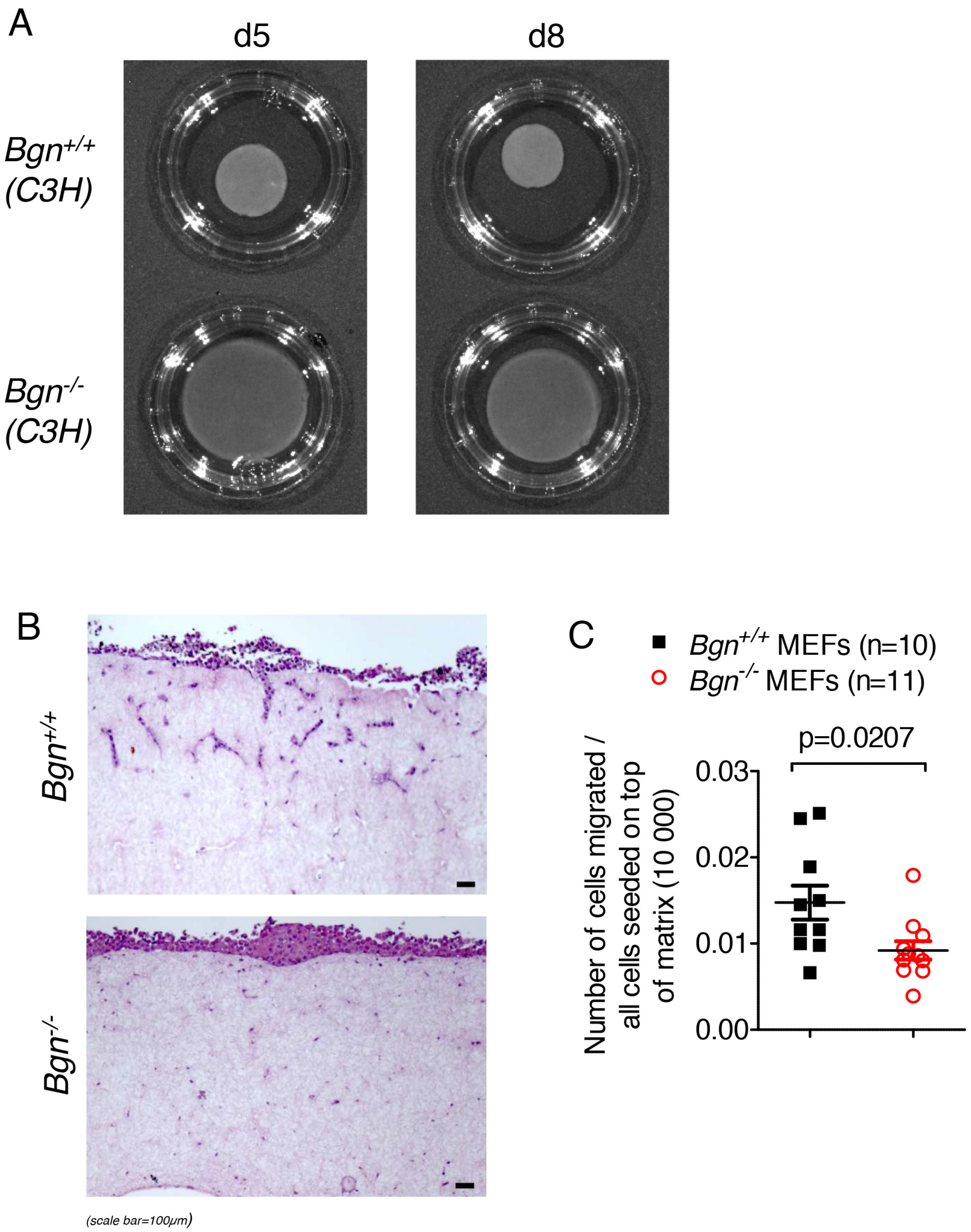

Supplementary Figure 3: Matrix contraction is dependent on *Bgn*$^{+/+}$ versus *Bgn*$^{-/-}$ MEFs on another genetic background. **(A)** Contraction of collagen matrices containing *Bgn*$^{+/+}$ or *Bgn*$^{-/-}$ MEFs as indicated for the respective groups. One representative collagen matrix per group on day 5 and day 8 of contraction is shown. **(B)** Representative images of histological slides of invasive cell migration in *Bgn*$^{+/+}$ matrices (top) versus *Bgn*$^{-/-}$ matrices (bottom) on day 7 of invasion. **(C)** Quantification of migrated cells per optical field (OF) related to 10 000 cells seeded on the top of the matrix. For each sample, cells in three different optical fields were counted and the average was calculated (*Bgn*$^{+/+}$ (n=10) versus *Bgn*$^{-/-}$ (n=11); p=0.0207).

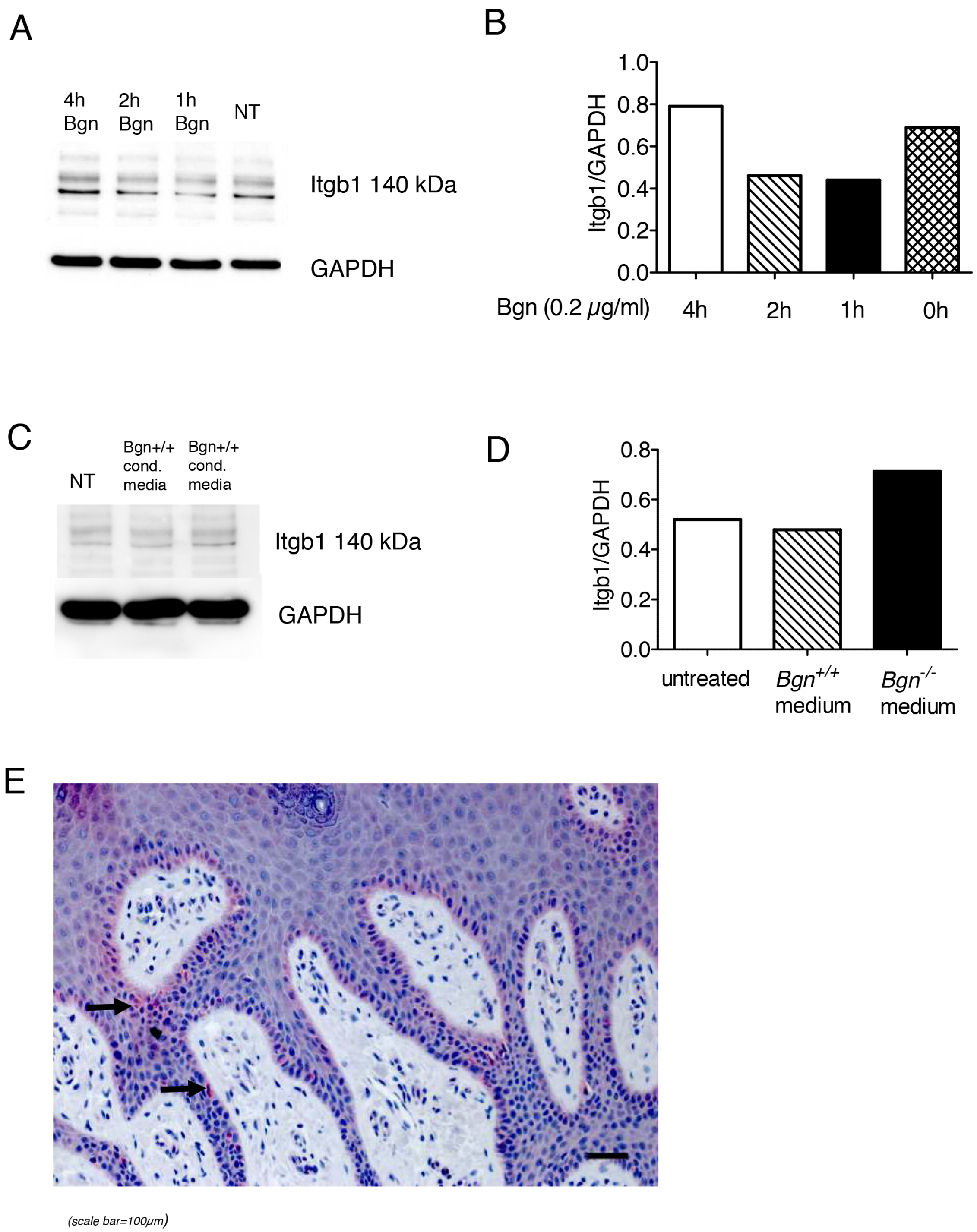

Supplementary Figure 4: Integrin-β1 expression in cells treated with biglycan and conditioned media of *Bgn*$^{+/+}$/*Bgn*$^{-/-}$ MEFs and in the normal skin. **(A)** A representative western blot analysis of Integrin-beta1 expression in B16 cells after exposing to 0.2 μg/ml soluble biglycan for different timepoints. GAPDH was used as control. **(B)** The bar diagram shows the quantification of integrin-β1 expression in B16 cells treated with Bgn for different timepoints, normalized to GAPDH expression. **(C)** B16 cells were exposed to the media from *Bgn*$^{+/+}$ and *Bgn*$^{-/-}$ MEFs conditioned for 12 hours. After 24 h exposure, cells were harvested and western blot analysis was performed. GAPDH was used as control. **(D)** The bar diagram shows the quantification of integrin-β1 expression in B16 cells treated with conditioned media from *Bgn*$^{+/+}$ and *Bgn*$^{-/-}$ MEFs, normalized to GAPDH expression. **(E)** Heathy skin tissue stained for integrin-β1 (red). Only cells of the basal layer of the epidermis express weakly integrin-β1 (black arrow).

Supplementary Table 1: Patients characteristics

Variable	Good prognosis (n=41)	Poor prognosis (n=40)
	% (absolute number)	% (absolute number
Pt. age median (range)	58.7 (35-83)	66.5 (30-93)
Gender		
female	48.8 (20)	35 (14)
male	51.2 (21)	65 (26)
Stage		
I	73.2 (30)	15 (6)
II	12.2 (5)	32.5 (13)
III	14.6 (6)	35 (14)
IV	0 (0)	17.5 (7)
